# The mitochondrial genome of the goosander (*Mergus merganser*) determined using next-generation sequencing

**DOI:** 10.1080/23802359.2019.1640080

**Published:** 2019-07-18

**Authors:** Seon-Mi Lee, Hye Sook Jeon, Jung A Kim, Jisoo Kim, Jungeun Park, Hyun-Jong Kil

**Affiliations:** a Animal Resources Division, National Institute of Biological Resources, Incheon, Republic of Korea;; b Environmental Health Research Department, National Institute of Environmental Research, Incheon, Republic of Korea

**Keywords:** Common merganser, goosander, mitochondrial genome, next generation sequencing

## Abstract

We report here the complete mitochondrial genome of *Mergus merganser*, which was determined using Illumina next-generation sequencing. The complete mitogenome is 16,631 bp, with 13 protein-coding genes, 22 transfer RNA genes, and 2 ribosomal RNA genes. The order and structure of the genes are similar to those of other Anatinae species. The overall base composition of the mitogenome is 28.7% (A), 21.8% (T), 16.0% (G), and 33.2% (C), with AT contents of 50.7%. Phylogenetic analysis based on 13 concatenated PCG sequences indicated that *M. merganser* is closely related to *M. squamatus* (HQ833701 and NC016723) with high bootstrap values (100%).

The goosander (*Mergus merganser*) is a large, piscivorous duck that has a crest of long head feathers like other species in the *Mergus* genus. Goosanders are mainly distributed across the Holarctic region and breed in tree cavities between March and May (Gregory et al. 1997). During the winter, goosanders usually migrate from their breeding sites to southern wintering areas to avoid zones where rivers and major lakes freeze (Keller 2009). Some goosanders migrate to rivers, lakes, or brackish water zones in South Korea, where they are considered to be transmitters of the Avian Influenza virus (NIER 2012). *Mergus merganser* is at risk due to threats stemming from pollution and habitat degradation and has shown reduced population sizes. To promote the conservation of *M. merganser* numbers, this species has been designated as a species of Least Concern in the IUCN Red List (BirdLife International 2018).

In this study, we determined the complete mtDNA sequence of *M.* merganser. The *M. merganser* tissue samples used in this study were collected from Guri-si, Gyeonggi-do, South Korea, numbered NIBR0000603491, and stored in the National Institute of Biological Resources (NIBR) in Incheon, South Korea. Total genomic DNA was extracted using the DNeasy Blood and Tissue Kit (Qiagen, Valencia, CA, USA) according to the protocol from the manufacturer. Next-generation sequencing was conducted with the Illumina HiSeq2500 platform at DNA LINK in Seoul, South Korea. The mitochondrial genome was de-novo assembled using CLC_assembler (ver. 4.010.83648, CLC QIAGEN). Assembly errors and gaps were manually corrected by paired-end WGS reads mapping by CLC_mapper (ver. 4.010.83648, CLC QIAGEN; Kim et al. 2015). Structural features and genes in the mitochondrial genome were predicted using Ge-seq (Tillich et al. 2017) and confirmed by DOGMA (Wyman et al. 2004) and ARWEN (Laslett and Canbäck 2008). The nucleotide sequences of the *M. merganser* mitogenome were deposited in GenBank under accession number MK862101.

The length of the complete mtDNA sequence is 16,630 bp, which is similar to other Anatinae species. The overall base composition for the mtDNA sequence is as follows: A (28.7%), T (21.8%), G (16.0%), and C (33.2%). The sequence has higher A-T content (50.7%) and lower G-C content (49.3%). In total, the sequence encodes 37 genes, of which 13 are protein-coding genes (PCGs), 22 are transfer RNA genes (tRNAs), 2 are ribosomal RNA genes (rRNAs), and 1 is a non-coding region (D-loop, 1059 bp) located between tRNA-glu and tRNA-phe. Among the 37 genes, the genes in *M. merganser* were distributed on the H-strand, except for the ND6 subunit gene and eight tRNA genes encoded on the L-strand.

In order to confirm the mitochondrial sequence obtained in this study, we selected an additional 28 individuals of 12 Anatinae species to reconstruct the phylogenetic tree generated in MEGA6 (Tamura et al. 2013); the common shelduck (*Tadorma tadorma*) was used as the out group. Our phylogenetic analysis, which was based on 13 concatenated PCG sequences (Figure 1), indicated that *M. merganser* is closely related to *Mergus squamatus* with high supporting bootstrap values (100%).

**Figure 1.**
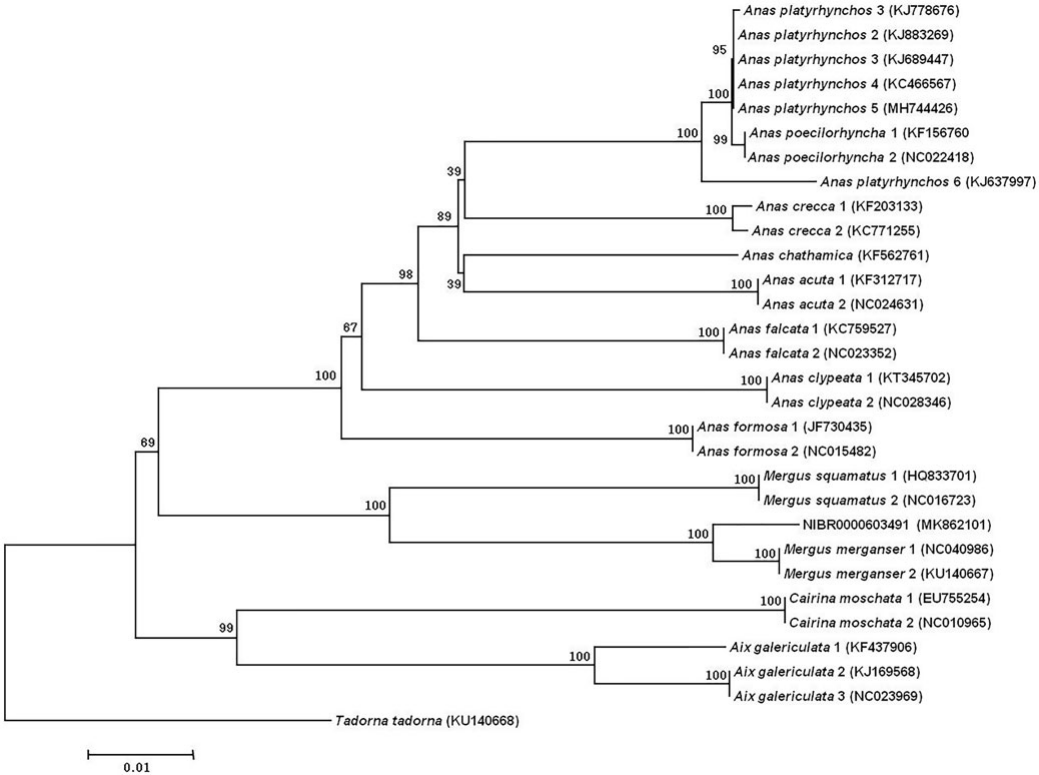
Neighbor-joining (NJ) phylogeny of Anatinae inferred from concatenated nucleotide sequences of the 13 protein-coding genes of the mitogenomes. Node labels indicate bootstrap values.
